# The β-1,3-Glucanase Degrades Callose at Plasmodesmata to Facilitate the Transport of the Ribonucleoprotein Complex in *Pyrus betulaefolia*

**DOI:** 10.3390/ijms24098051

**Published:** 2023-04-29

**Authors:** Yunfei Yu, Shengyuan Wang, Chaoran Xu, Ling Xiang, Wenting Huang, Xiao Zhang, Baihui Tian, Chong Mao, Tianzhong Li, Shengnan Wang

**Affiliations:** Laboratory of Fruit Cell and Molecular Breeding, China Agricultural University, Beijing 100193, China

**Keywords:** β-1,3-glucanase, long-distance movement, ribonucleoprotein complex, PbANK, *Pyrus betulaefolia*, callose deposition

## Abstract

Grafting is widely used to improve the stress tolerance and the fruit yield of horticultural crops. Ribonucleoprotein complexes formed by mRNAs and proteins play critical roles in the communication between scions and stocks of grafted plants. In Pyrus betulaefolia, ankyrin was identified previously to promote the long-distance movement of the ribonucleoprotein complex(*PbWoxT1-*PbPTB3) by facilitating callose degradation at plasmodesmata. However, the mechanism of the ankyrin-mediated callose degradation remains elusive. In this study, we discovered a β-1,3-glucanase (EC 3.2.1.39, PbPDBG) using ankyrin as a bait from plasmodesmata by co-immunoprecipitation and mass spectrometry. Ankyrin was required for the plasmodesmata-localization of PbPDBG. The grafting and bombardment experiments indicated that overexpressing PbPDBG resulted in decreased callose content at plasmodesmata, and thereby promoting the long-distance transport of the ribonucleoprotein complex. Altogether, our findings revealed that PbPDBG was the key factor in ankyrin-mediated callose degradation at plasmodesmata.

## 1. Introduction

Grafting is an important cultivation technique in agricultural production. Furthermore, the interaction between grafted rootstock and scion often leads to improved stress resistance and increased fruit quality and yield [1,2,3]. Numerous studies have shown that diverse macromolecules such as mRNAs are transported over long-distances between rootstock and scion through the phloem. For instance, the mRNAs of two auxin-responsive genes *IAA18* and *IAA28* were synthesized in mature leaves of *Arabidopsis thaliana* and transported long-distance through the phloem to regulate lateral root development [4]. The more in-depth studied Gibberellic acid insensitive (*GAI*) mRNA, which is characterized by a highly conserved N-terminal DELLA domain, can move from the rootstock to the scion to dwarf the apple plants [5]. In addition, the mRNAs *FT*, *BEL5* and *PbDRM* were all shown to be mobile, resulting in regulating flowering, tuber yields and drought resistance, respectively [1,6,7].

The loading recognition, the transport in phloem, and the unloading to the target cell of long-distance transported RNAs are depending on forming a stable ribonucleoprotein complex with specific RNA-binding proteins [8,9]. The polypyrimidine tract binding (PTB) protein CmRBP50 was identified as the first ribonucleoprotein complex by co-immunoprecipitation in the phloem sap of pumpkin. CmRBP50 recognizes mRNAs via the CUCU motifs [10]. Its homologous proteins in *Solanum tuberosum*, StPTB1 and StPTB6, bind to the 3′UTR region of *StBEL5* mRNA and facilitate its long-distance transport in phloem, thereby improving potato tuber yield [11]. PbPTB3 (polypyrimiding tract-binding), the homologous protein of CmRBP50 in pear, forms an RNP complex with the wuschel-related homeobox transport1(*PbWoxT1*) mRNA to assist its long-distance transport in phloem through the graft union, affecting the style length of scion [12]. To facilitate the transport of the *PbWoxT1-*PbPTB3 RNP complex, a WD40 protein transpareet testa glabra1(PbTTG1) bind with PbPTB3 to increase the stability of the binding between PbPTB3 protein and *PbWoxT1* mRNA. Furthermore, PbANK, which is an ankyrin repeat containing protein, degradate the callose at plasmodesmata (PD) to facilitate the long-distance trafficking [2,3]. However, it remains elusive how this ribonucleoprotein complex is transported cell-to-cell through plasmodesmata and finally loaded into phloem.

Plasmodesmata are channels between cells for the exchange of endogenous macromolecules and the spread of exogenous pathogens [13]. Plasmodesmata only permits the passage of specific molecules smaller than the size of exclusion limit (SEL). The SEL is generally about 10~60 kDa and varies depending on the plant species, the organ types, and the developmental stages [14,15]. Callose is deposited in the cell walls of the outer regions at the two ends of the plasmodesmata [16,17,18,19,20]. Callose is a linear homopolymer formed by glucose residue through β-1,3-linkages and sometimes β-1,6-branches using UDP-glucose as a substrate by callose synthase [21,22,23,24,25]. The deposition and degradation of callose is a dynamic process, which plays a decisive role in the opening and closing state of plasmodesmata, directly affecting the plasmodesmata permeability and regulating the transport efficiency of biological macromolecules and pathogens [26,27,28]. It was reported that callose deposition at plasmodesmata provided a physical barrier limiting or preventing the spread of virus in the resistant and local-lesion hosts [29].

Callose is degraded by glucan endo-1,3-beta-glucosidases (beta-1,3-glucanases; 1,3-beta-D-glucan glucanohydrolases, EC 3.2.1.39), which belong to the PR-2 class of proteins that can break the β-1,3-glycosidic bonds [30,31]. β-1,3-glucanases have been implicated in phloem transport, cell wall biosynthesis and several other developmental processes [32,33,34,35,36], and they may also play a direct role in the plant’s defense against fungal pathogens [37]. So far, various β-1,3-glucanase genes from *Arabidopsis thaliana* [38], *Oryza sativa* [39], *Triticum aestivum* [40], and *Zea mays* [41] have been identified to be related to the pathogen resistance of the host. In a β-1,3-glucanase (GLU I)-deficient mutant (TAG4.4) of tobacco, the cell-to-cell movement of tobacco mosaic virus (TMV) was delayed and the SEL of the plasmodesmata was also reduced from 1.0 to 0.85 nm coupled with the increased deposition of callose [26]. It is well-studied how callose deposition is regulated upon pathogen infection. However, it remains obscure whether similar mechanisms control the long-distance transport of the ribonucleoprotein complexes.

In this study, we discovered and characterized a novel plasmodesmata-localized β-1,3-glucanase (PbPDBG) in *Pyrus betulaefolia* that can interact with PbANK and facilitate the transport of the *PbWoxT1-*PbPTB3 ribonucleoprotein complex. The PbPDBG can directly degrade the callose at plasmodesmata to open the plasmodesmata channel, thereby promoting the intercellular movement of PbPTB3, and further improving the long-distance transport of *PbWoxT1* mRNA. Our findings identified the role of PbPDBG in mediating the movement of the ribonucleoprotein complex, which provided unprecedent insights into the mechanism of ribonucleoprotein complex transport in phloem.

## 2. Results

### 2.1. Screening and Identification of the PbANK Interacting Proteins

**Figure 1:**
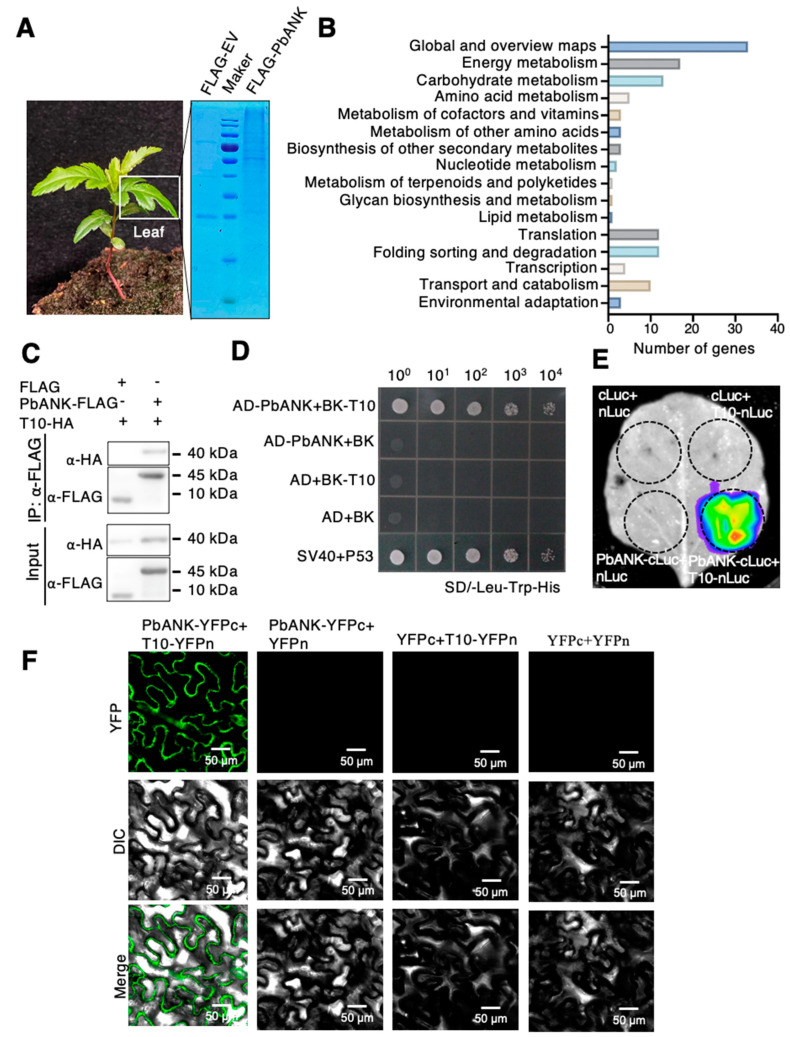
The PbANK-interacting proteins at plasmodesmata. (**A**) The PbANK-interacting proteins were co-immunoprecipitated from leaves of *Pyrus betulaefolia* using PbANK-FLAG. The total precipitated proteins were visualized by Coomassie staining. (**B**) KEGG enrichment analysis of proteins interacted with PbANK (FDR < 0.01). (**C**) The interaction between AD-PbANK and BK-T10 was analyzed by co-immunoprecipitation analysis. (**D**) The interaction between AD-PbANK and BK-T10 was analyzed by yeast two-hybrid analysis. SV40-AD and P53-BK were used as positive controls. AD and BK empty vectors were used as negative controls. (**E**) Luciferase completion imaging analysis the interaction between cLuc-PbANK and nLuc-T10. Empty vectors with cLuc and nLuc were used as negative controls. (**F**) The interaction between YFPc-PbANK and YFPn-T10 was analyzed using bimolecular fluorescence complementation in *N. benthamiana* epidermal cells. YFPc and YFPn empty vectors were used as negative controls. Bars, 50 μm.

To explore how PbANK affects the intercellular diffusion of the ribonucleoprotein complex formed by the *PbWoxT1* mRNA and the PbPTB3 protein, PbANK-FLAG fusion protein was employed as a bait in the co-immunoprecipitation/mass spectrometry (Co-IP/MS) analysis of the plasmodesmata proteins extracted from the leaves of *P. betulaefolia* compared with using empty FLAG-tag as a bait (Figure 1A and Figure S1A). In total, 67 proteins were identified as specific interaction partners of PbANK at plasmodesmata by MS (Figure S1B and Table S2). GO and KEGG enrichment analysis performed on these 67 candidates showed that the potential PbANK-interacting proteins were involved in a variety of functions, including biological processes, cellular component and molecular function (Figure 1B and Figure S1C). Worth noting that 11 proteins were enriched in the transport-related pathways (Table 1), among which the Transport related protein 10 (T10) shared more than 90% similarity with β-1,3-glucanase, a gene responsible for callose degradation, in other species by sequence alignment analysis (Figure S2).

To further study the interaction between T10 and PbANK, PbANK-FLAG and T10-HA fusion protein were co-transformed into *N. benthamiana* to perform Co-IP analysis using an anti-HA monoclonal antibody. The result showed that T10-HA could co-immunoprecipitated with PbANK-FLAG (Figure 1C). Furthermore, yeast-two-hybrid, BiFC and LCI analysis were performed to validate the interaction. The coding sequence of T10 was inserted into the pGBKT7 vector and co-transfected into yeast with the pGADT7-PbANK fusion vector. The empty vectors of pGADT7 and pGBKT7 were used as negative controls, and P53 and SV40 were used as positive controls. The results showed that the pGADT7-PbANK and pGBKT7-T10 co-transformed yeast strains could grow normally on SD/-Leu-Trp-His dropout medium, while the negative control could not survive (Figure 1D). The Luciferase complementation imaging (LCI) experiment confirmed that T10 interacted with PbANK (Figure 1E). Finally, PbANK-YFPc fusion vector was co-infiltrated with T10-YFPn in *N. benthamiana* leaf for BiFC analysis. Compared to different negative controls, only co-infiltration of PbANK-YFPc and T10-YFPn showed fluorescence signals (Figure 1F). In conclusion, four different methods verified the interaction between T10 and PbANK. Since the main function of β-1,3-glucanase is to degrade callose, we speculate that T10, which shared more than 90% sequence similarities with identified β-1,3-glucanase (Figure S2), is likely involved in regulating the PbANK-mediated intercellular diffusion of ribonucleoprotein complexes.

### 2.2. Characterization of the PbANK Interaction Protein PbPDBG

**Figure 2:**
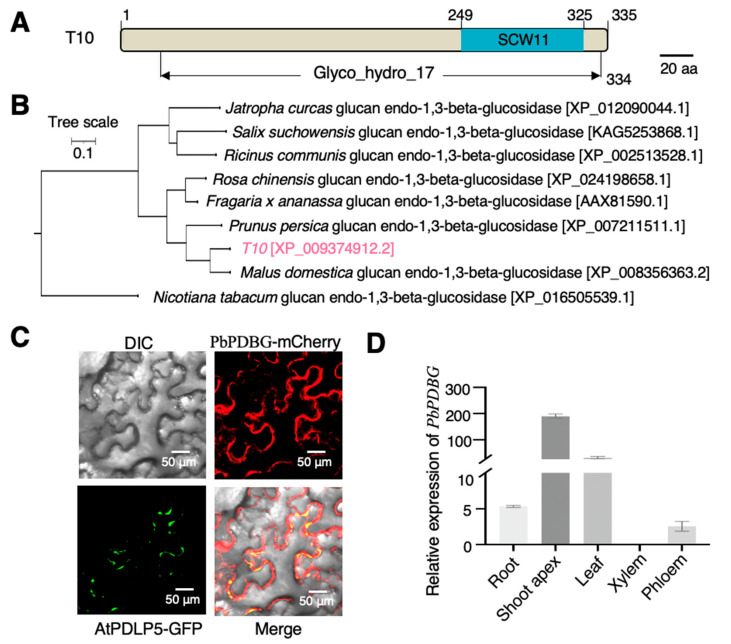
Characterization of the PbPDBG. (**A**) Conserved domain analysis of T10 protein. The numbers represent amino acid positions. The blue box marks the SCW11 domain. The scale bar stands for 20 amino acids. (**B**) Phylogenetic tree shows the clustering analysis of *T10* (XP_009374912.2) gene of *Pyrus betulaefolia* with homologous sequences from other plants. (**C**) Subcellular co-localization analysis of PbPDBG-mCherry. The AtPDLP5-GFP fusion protein was used as the plasmodesmata marker. Red signal represents PbPDBG, green signal represents AtPDLP5. The scale bars were 50 μm. (**D**) RT-qPCR analysis for the expression level of *PbPDBG* in different tissue parts of *Pyrus betulaefolia*. *PbActin* was used as the internal control.

To further confirm the identity of T10, the full-length 335 amino acids sequence of T10 was analyzed in the National Center for Biotechnology Information database. T10 shared 99% similarity with the β-1,3-glucanase (XP_009374912.2) belonging to the Glycosyl hydrolases family 17, which contained the conserved functional domain of SCW11 (Figure 2A). Phylogenetic analysis showed that T10 was the closest to the β-1,3-glucanase of *Malus domestica* (Figure 2B). Through phylogenetic analysis, conserved domain analysis and sequence similarly analysis, we defined T10 protein as a β-1,3-glucanase in pear. In order to clarify the localization of the β-1,3-glucanase T10 and the relationship with the plasmodesmata, T10-mCherry fusion was constructed for co-localization analysis, together with the plasmodesmata marker fusion AtPDLP5-GFP [42] driven by *CaMV 35S* promoter. The result showed that the T10 is co-localized with the plasmodesmata marker AtPDLP5 (Figure 2C). Based on the location and function, T10 was named *PbPDBG* (*Pyrus betulaefolia* plasmodesmata-localized β-1,3-glucanase). Tissue-specific expression in *P. betulaefolia* showed that *PbPDBG* was expressed mainly in the shoot tips and leaves, but hardly in the xylem (Figure 2D). The above results indicated that *PbPDBG* localized at plasmodesmata and played a possible role in every tissue except xylem.

### 2.3. PbPDBG Regulated Callose Deposition at Plasmodesmata

**Figure 3:**
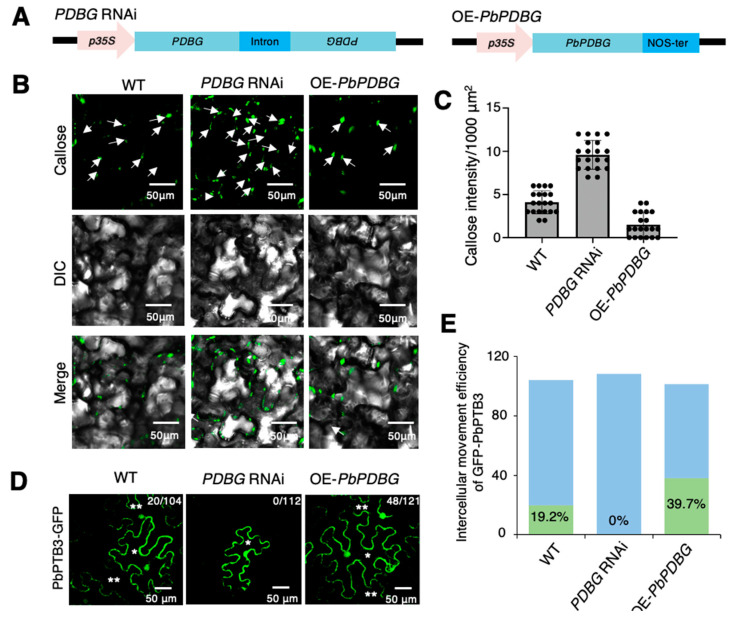
PbPDBG regulated callose deposition and intercellular movement of PbPTB3-GFP. (**A**) The diagram of vectors used for overexpressing PbPDBG and PDBG RNAi. (**B**) Leaves of *N. benthamiana* overexpressing PbPDBG, PDBG RNAi and wild-type (WT) *N. benthamiana* were stained with aniline blue to reveal callose content. The scale bars were 50 μm. The white arrows point at callose. (**C**) The quantity analysis of callose in OE-PbPDBG, PDBG RNAi and wild type *N. benthamiana* leaves per 1000 μm^2^. The black dots represent the amount of callose observed in each sample, and error bars represent the standard deviation. (**D**) The intercellular movement of PbPTB3-GFP in wild-type (WT), PDBG RNAi and PbPDBG overexpressing leaves. The numerator of the fraction in the up right corner represented the number of cells in which PbPTB3-GFP was moved, and the denominator represented the total number of cells bombarded. The ‘*’ indicate the bombarded cells, and the ‘**’ indicate the cells with spread GFP signal. The scale bar was 50 μm. (**E**) Statistical analysis of the intercellular mobility efficiency of PbPTB3-GFP in (**D**). Blue bars represent bombarded cells. Green bars represent cells with spread GFP signal.

In previously studies, β-1,3-glucanases have been found to degrade callose, whose component is glucan [43]. To further demonstrate the function of PbPDBG on callose degradation, *PbPDBG* overexpression (OE-*PbPDBG*) and *PDBG* silencing (*PDBG* RNAi) stable transgenic lines were generated using pFGC5941 vector driven by *35S* promotor in *N. benthamiana*, in which the expression level of *PDBG* was successfully overexpressed or silenced (Figure 3A and Figure S3A). The β-1,3 glucanase activities of OE-PbPDBG, PDBG RNAi and wild-type (WT) leaves of N. benthamiana were determined respectively, and the β-1,3 glucanase activity of OE-PbPDBG tobacco strain were the highest. The β-1,3 glucanase activity of PDBG-silenced tobacco was significantly lower than that of the wild type (Figure S3B).The callose deposited in *PDBG* RNAi line was significantly higher than that in WT, while the callose deposition of OE-*PbPDBG* was significantly lower than that in WT (Figure 3B,C). In conclusion, PbPDBG is a protein that localized at plasmodesmata and associated with callose deposition.

The content of callose directly affects the permeability of the plasmodesmata. In order to test whether the intercellular movement of PbPTB3 depends on the degradation of callose by PbPDBG, the PbPTB3-GFP fusion expression vector was bombarded into single cells of OE*-PbPDBG*, *PDBG* RNAi and wild-type (WT) leaves of *N. benthamiana*. The results showed that a total of 121 single OE*-PbPDBG* leaf cells were successfully bombarded with the PbPTB3-GFP protein spread to 48 neighboring cells, indicating a 39.7% mobile efficiency. That was much higher than 22 spreads from 104 bombarded cells with a 19.2% mobile efficiency in the WT control. In contrast, a total of 112 single *PDBG* RNAi leaf cells were successfully bombarded with no PbPTB3-GFP detected in the neighboring cells (Figure 3D,E). In conclusion, these results indicated that PbPDBG could promote the intercellular movement efficiency of PbPTB3-GFP.

### 2.4. PbANK Regulated the Localization of PbPDBG

**Figure 4:**
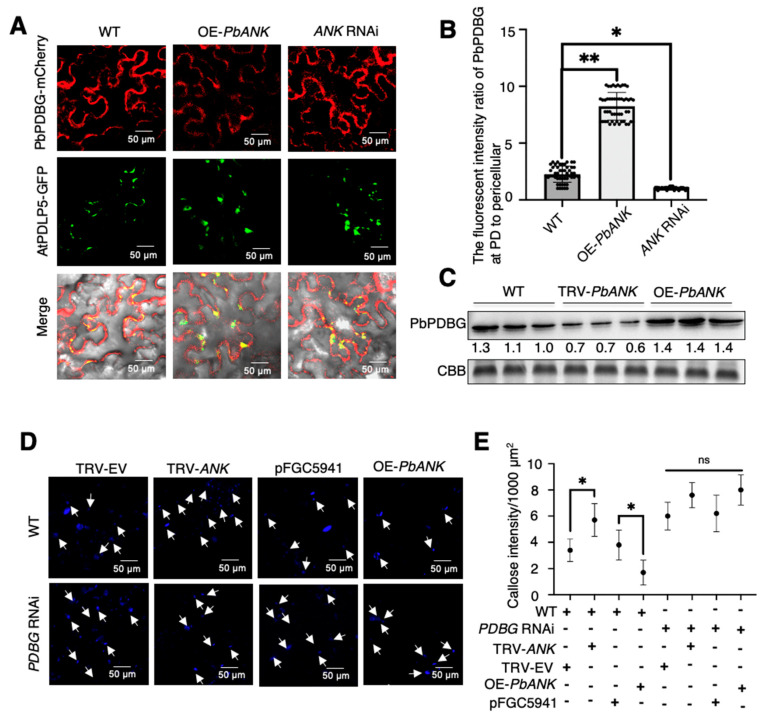
PbANK recruited PbPDBG to the plasmodesmata. (**A**) Subcellular localization of PbPDBG−mCherry in *N. benthamiana* leaves from wild−type, the line overexpressing *PbANK* and silencing *ANK*. Red signal represents PbPDBG, green signal represents AtPDLP5.The scale bars were 50 μm. (**B**) Image J was used to measure the fluorescence intensity of PbPDBG−mCherry at the plasmodesmata and the pericellular. More than 50 fields were observed for each treatment. Error bars represent the standard deviation (*, *p* < 0.05; **, *p* < 0.01). (**C**) Western blot analysis was performed on the total proteins from plasmodesmata using the α-β-1,3-glucanase monoclonal antibody in wild−type, *PbANK*−overexpression lines and *PbANK* silencing lines in *Pyrus betulaefolia*. The relative expression levels were normalized to the amount of total protein stained by coomassie brilliant blue (CBB). (**D**) The callose deposition in leaves of WT and *PDBG* RNAi *N. benthamiana* with *PbANK* overexpression, or *ANK* silencing. pFGC5941 and TRV empty vector was used as control. The callose was stained with aniline blue. The scale bars were 50 μm. The white arrows point at callose. (**E**) The amount of callose per 1000 μm^2^ was observed and counted under a confocal microscope (*, *p* < 0.01; ns, no significance). More than 50 fields were observed for each treatment.

Furthermore, in order to explore the regulatory relationship between PbANK and PbPDBG at plasmodesmata, PbPDBG-mCherry fusion protein driven by *35S* promoter was transiently overexpressed in the stable overexpression line OE-*PbANK* and the stable *ANK* silencing line (*ANK* RNAi) of *N. benthamiana* generated previously (Figure S4A) [3]. The results showed that in WT line, PbPDBG-mCherry was mainly localized in the cytoplasm and plasmodesmata. Overexpressing PbANK promoted plasmodesmata localization of PbPDBG, while silencing *ANK* resulted in the retention of PbPDBG in the cytoplasm (Figure 4A). The PbPDBG fluorescence intensity located at plasmodesmata was analyzed by Image J. The localized fluorescence of PbPDBG was concentrated at PD in OE-*PbANK* while was dispersed in *ANK* RNAi lines compared to WT (Figure 4B). In order to further analyze the effect of PbANK on PbPDBG accumulation at plasmodesmata, plasmodesmata protein was extracted from *P. betulaefolia* transiently overexpressing *PbANK* (OE-*PbANK*) or silencing *PbANK* (TRV-*PbANK*). Western blot analysis showed that overexpressing *PbANK* (OE-*PbANK*) increased the content of PbPDBG at plasmodesmata, while silencing *PbANK* (TRV-*PbANK*) decreased the level of PbPDBG at plasmodesmata compared to WT (Figure 4C).

According to the above results and our previous studies, PbANK and PbPDBG can reduce the content of callose [3]. We next investigated the regulatory relationship between PbANK and PbPDBG in the process of reducing callose content. The callose content was analyzed in the stable silencing line of *PDBG* (*PDBG* RNAi), *PbANK* transiently overexpressing line (OE-*PbANK*) and *ANK* silencing line with *TRV* vector (TRV-*ANK*) through VIGS system (Figure S4B,C). The results showed that the callose content was significantly increased in TRV-*ANK* while strikingly lower in OE-*PbANK* compared to the control in WT. The callose content in *PDBG* RNAi lines was higher than that in WT and not affected by the *ANK* expression level (Figure 4D,E). These results demonstrated that PbPDBG could be recruited to the plasmodesmata by PbANK to degrade callose.

### 2.5. PbPDBG Facilitated the Long-Distance Transport of the Ribonucleoprotein Complex

**Figure 5:**
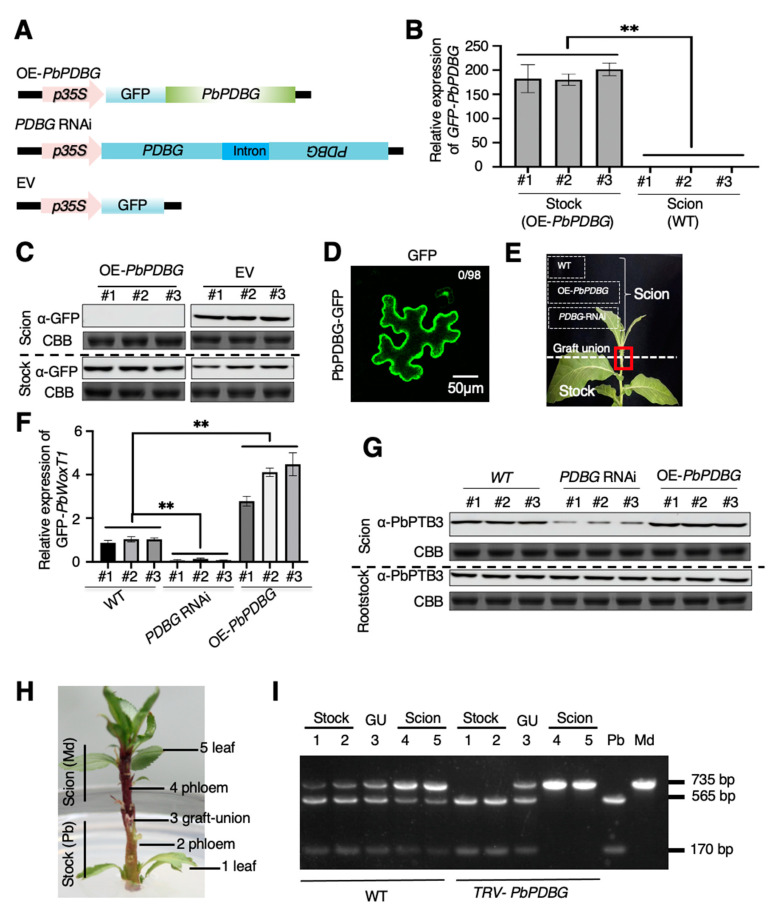
PbPDBG facilitated long-distance transport of the ribonucleoprotein complex. (**A**) The vector diagrams of OE-*PbPDBG*, *PDBG* RNAi and empty vector used for identifying the mobility of PbPDBG (**B**) RT-qPCR analysis of *PbPDBG* in WT/OE-*PbPDBG* grafting system. The results showed the mean ± SD derived from three technical replicates. Data were analyzed using Student’s *t*-test (**, *p* < 0.01). (**C**) Western blot analysis using the α-GFP monoclonal antibody in WT/OE-*PbPDBG* and WT/EV grafting system. The relative expression levels were normalized to the amount of total protein stained by Coomassie brilliant blue (CBB). (**D**) The intercellular movement of PbPDBG-GFP in *N. tabacum* leaves. The numerator of the fraction in the up right corner represented the number of cells in which PbPDBG-GFP was moved, and the denominator represented the total number of cells bombarded. The scale bar was 50 μm. (**E**) Photograph representing wild type, OE-*PbPDBG* and *PDBG* RNAi scion grafted onto PbPTB3-mCheery and *PbWoxT1*-GFP co-transgenic *N. tabacum* stocks. The red box marks the graft union. (**F**) RT-qPCR analysis of the relative expression of *PbWoxT1-GFP* in different scions in (**E**). The results show the mean ± SD derived from three technical replicates. Data were analyzed using Student’s *t*-test (**, *p* < 0.01). (**G**) Western blot analysis using the α-PbPTB3 polyclonal antibody in different grafting system in (**E**). The relative expression levels were normalized to the amount of total protein stained by Coomassie brilliant blue (CBB). (**H**) A grafting system with *P. betulaefolia* (Pb) as rootstock and *M. domestica* (Md) as scion. (**I**) dCAPS RT-PCR analysis of *WoxT1* expression in a *M. domestica* (scion)/*P. betulaefolia* (rootstock) micrograft silencing *PbPDBG*. The different tissues from the micograft were indicated by numbers shown in (**H**). Md, *Malus domestica* after endonuclease *SfcI* digest; Pb, *Pyrus betulaefolia* after endonuclease *SfcI* digest; GU, graft union.

Since PbPDBG promoted the intercellular movement of PbPTB3 through degrading callose, it became our concern whether PbPDBG protein could be transported along with PbPTB3. In order to explore whether PbPDBG protein was mobile, OE-*PbPDBG*, *PDBG* RNAi stable transgenic lines in *N. tabacum* were generated (Figure 5A and Figure S5A). WT was used as scion to graft on OE*-PbPDBG* stocks or the rootstocks expressing mobile GFP [15]. The stem segments of rootstock and scion 2 cm from the grafting interface were collected separately to extract total protein and RNA. RT-qPCR analysis revealed that there was *PbPDBG-GFP* expression in OE*-PbPDBG* stocks, while absolutely not in WT (Figure 5B). Western blot showed that the fusion protein PbPDBG-GFP could be expressed in stock but could not be transported to the WT scion, while the mobile GFP protein could be detected in the WT scion (Figure 5C). Meanwhile, we overexpressed *PbPDBG-GFP* in *N. benthamiana* single cells by bombardment. There was absolutely no intercellular diffusion in 98 bombarded cells (Figure 5D). These results suggested that *PbPDBG* mRNA and its encoded protein were not mobile.

According to the above results, PbPDBG facilitated the intercellular movement of PbPTB3, which could bind to *PbWoxT1* mRNA for its long-distance transport. To confirm PbPDBG affecting the long-distance transport efficiency of the ribonucleoprotein complex formed by PbPTB3 and *PbWoxT1* mRNA, a *pSUC2::PbPTB3-mCherry* and *p35S::PbWoxT1-GFP* co-transfected *N. tabacum* was used as stock to graft OE*-PbPDBG*, *PDBG* RNAi and WT (Figure 5E). One month after grafting, the total RNA from the scion leaf was extracted to detect the relative expression of *PbWoxT1-GFP*. The *PbWoxT1-GFP* mRNA expression detected in the scion of *PDBG* RNAi *N. tabacum* was significantly lower than that in WT, while that was significantly higher in the scion of OE*-PbPDBG* (Figure 5F). At the same time, western blot performed on the total protein extracted from the scion leaf showed that the expression of PbPTB3-mCherry from the stock could detected in the scion, with the level significantly higher in the grafted scion from the OE*-PbPDBG* lines and significantly lower from the *PDBG* RNAi lines (Figure 5G).

To further confirm the effect of *PbPDBG* on long-distance transport of ribonucleoprotein complex, *M. domestica* as scion was grafted on the *P. betulaefolia* stock (Figure 5H). VIGS was used to silence *PbPDBG* expression in the grafting system through infiltrating *pTRV1-EV* and *pTRV2-PbPDBG* using agrobacterium (Figure S5B,C). At 30 days after grafting, each tissue was collected to extract total RNA for verifying the mobility of *PbWoxT1* using dCAPS (Figure 5H). A 735 bp PCR product of *WoxT1* mRNA was cloned in *P. betulaefolia* and *M. domestica* using dCAPs primers. *PbWoxT1* can be cleaved into 565 bp and 170 bp segments by *SfcI*, while *MdWoxT1* cannot. The *WoxT1* PCR product of each tissue from the stock and scion were purified and digested with *SfcI*. There were three bands at 735 bp, 565 bp and 170 bp in both scion and stock of the WT heterografts. However, cleavage products were not detected in the scion of the *PbPDBG* silencing line (Figure 5I). Altogether, these results indicated that *PbPDBG* played essential roles in the long-distance transport of the ribonucleoprotein complex formed by *WoxT1* mRNA and PbPTB3.

## 3. Discussion

Our previous research revealed the role of PbANK in facilitating the long-distance movement of *PbWoxT1* mRNA mediated by PbPTB3. In order to identify the downstream regulatory genes of PbANK, Co-IP/MS analysis was used to screen proteins that could interact with PbANK. We identified PbPDBG, a member of the glycosyl hydrolase family, involved in the degradation of sugar under anaerobic or anoxic conditions [44,45]. We speculated that PbPDBG, as a downstream regulatory protein of PbANK, facilitated the intercellular movement of *PbWoxT1* mRNA in coordination with PbPTB3 by degrading callose. Subcellular localization of PbPDBG showed that it could co-localize with AtPDLP5-GFP at plasmodesmata (Figure 2C). In addition, tissue-specific analysis showed that PbPDBG was mainly and highly expressed in stem tip, leaf and root (Figure 2D), which are important source and sink organs for loading and unloading of macromolecules through plasmodesmata [2,3]. Callose is the gate of plasmodesmata channel [28,43,46]. β-1,3-glucanase accumulated in stem tip, leaf and root to regulate the macromolecules exchange between source and sink organs by degrading callose. These were also consistent with the previous study on cell-to-cell movement of *Potato virus X* [47].

Plant endogenous mRNAs should bind to specific proteins to form ribonucleoprotein complexes which could be transported cell-to-cell and then loaded into phloem for long-distance trafficking. In this process, many proteins are involved in facilitating transport. The intercellular transport of plant endogenous mRNAs and then long-distance transport in phloem is similar to the process of virus diffusion in plants. We hypothesized that the mechanism of ribonucleoprotein complex diffusion cell-to-cell may be similar to that of virus. β-1,3-glucanase is widely distributed in higher plants, including pollen tube wall, sperm cell wall and sieve tube end wall, playing important roles in plant growth and development [48,49,50]. Previous studies have shown that, when the virus infects plants, GLU I deficient mutant exhibits restricted intracellular trafficking of the virus movement protein (MP), thus inhibiting the further spread of the virus [26]. High expression of beta-1,3-glucanase can degrade callose to promote the virus infection of plants [31,51]. In this study, we found that PbPDBG can degrade callose and enhance the intercellular movement efficiency of PbPTB3 (Figure 3). This suggests that PbPDBG does play a facilitating role in the cell-to-cell movement of ribonucleoprotein complexes, similar to the mechanism of virus diffusion.

Similar events take place in the phloem of perennials in which the sieve-plate pores become closed in autumn by the deposition of β-1,3-glucan, and reopen in spring by activation of β-1,3-glucanase [52]. In our previous studies, we have demonstrated that the *PbWoxT1*-PbPTB3-formed ribonucleoprotein complex can be transported over long-distances between rootstock and scion via phloem [12]. In this research, it was confirmed that PbPDBG facilitated the long-distance transport of *PbWoxT1*-PbPTB3 complex in phloem (Figure 5). It requires further investigation whether PbPDBG is also involved in callose degradation in sieve-plate pores of phloem.

In all our studies, we found that both PbANK and PbPDBG affect the content of callose (Figure 3B) [3]. The functional analysis showed that PbANK did not have the ability to directly degrade callose, but relied on recruiting the PbPDBG [26]. Indeed, PbANK level could not significantly affect the content of callose when *PbPDBG* was silenced (Figure 4D). But overexpressed and silenced *PbANK* significantly changed the expression level of PbPDBG in plasmodesmata protein of *P. betulaefolia* (Figure 4C). Therefore, we hypothesized that PbANK-mediated callose degradation may be through the recruitment of PbPDBG to PD. In addition, the subcellular localization of PbPDBG in OE-*PbANK* and *ANK* RNAi showed that the distribution of PbPDBG was significantly more concentrated at plasmodesmata in OE-*PbANK* lines (Figure 4A), indicating that PbPDBG aggregated to PD under the control of PbANK. In previous studies, we also demonstrated that PbANK interacted with PbPTB3 and mediated ribonucleoprotein complex trafficking through callose degradation [3]. Thus, we speculate that PbANK may act as an intermediate bridge when the *PbWoxT1-*PbPTB3 formed ribonucleoprotein complex enters the phloem through plasmodesmata. However, the specific adjustment mechanism between the three still needs further in-depth studies.

In the process of screening the downstream regulatory genes of PbANK, we found that, in addition to PbPDBG, PbANK can also interact with ferritin, monohydroascorbate reductase, etc. (Table 1). Ferritin is a functional protein that stores iron in plants, animals and microorganisms. It exists in plastids and can maintain the dynamic balance of iron. In addition, Ferritin gene is significantly up-regulated in response to biotic and abiotic stresses, such as disease and drought, to improve stress tolerance [53]. Like Ferritin, Monohydroascorbate reductase gene can also enhance the ability of plants to withstand high temperature, low temperature and diseases [54,55]. As a multifunctional protein, it remains to be further explored whether PbANK participates in the regulatory pathways of Ferritin and Monodehydroascorbate reductase, and whether PbANK plays a role together with them to form protein complexes. These will also be our future research directions, which can enrich our understanding the mechanism of intercellular and long-distance transport of macromolecules.

## 4. Materials and Methods

### 4.1. Plant Materials and Growth Conditions

The seedlings of *Pyrus betulaefolia* and *Malus domestica* were grown in the nursery of China Agricultural University. The *P. betulaefolia* seeds were washed with water and incubated in dark and humid environment at room temperature for 3–5 days for germination, followed by transfer to the soil at 24 °C with 16 h/8 h (light/dark) photoperiod and a relative humidity of >85%. After 5–6 weeks of growth, it was used for *Agrobacterium* EH105 infection. The *N. benthamiana* seedlings were directly cultured in the soil under the same condition as indicated above. After 3–4 weeks of growth, it was used for *Agrobacterium* infection tests.

Tissue-cultured apple, pear and tobacco (*Nicotiana benthamiana* of wild type, *Nicotiana tabacum* var. Wisconsin 38 of wild type, *PbPTB3* and *PbWoxT1* co-transgenic lines, *PbANK* overexpression and silencing lines and *PDBG* overexpression and silencing lines) were cultured in MS medium under the same growth conditions as described in [19]. After growing in MS medium for 3–4 weeks, tobacco was moved into soil in seedling room for another 2–3 weeks and used for *Agrobacterium* infection and grafting. All plant were cultured at 24 °C, a relative humidity of 85% in a 16-h light:8-h dark photoperiod under a light intensity of 100 μmol m^−2^ s^−1^ provided by cool-white fluorescent tubes.

### 4.2. Conserved Domain and Phylogenetic Analysis

The amino acid sequence of PbPDBG (SUB13117513) was analyzed in NCBI (https://www.ncbi.nlm.nih.gov/Structure/cdd/wrpsb.cgi, accessed on 17 April 2023) for conserved domain analysis. The homologous sequences of PbPDBG in other species were obtained from NCBI (http://www.ncbi.nlm.nih.gov/BLAST, accessed on 17 April 2023). MEGA5 was used for phylogenetic analysis using the Neighbor-Joining method. The evolutionary distances were computed according to the JTT matrix-based method [3].

### 4.3. RNA Extraction, Reverse Transcription, RT-qPCR Analysis

The total RNA of *P. betulaefolia*, *M. domestica* and tobacco was extracted with RNAprep Pure Plant Plus Kit (Polysaccharides and Polyphenolics rich) from Tiangen Biotech company, according to the manufacturer’s instructions. To avoid genomic DNA contamination, extracted RNA was treated with DNase I. RNA was reverse transcribed into cDNA by the Aidlab Bio’s TRUESCript 1st Strand cDNA Synthesis Kit. RT-qPCR assay of all tissues was carried out on Thermo Fisher Scientific’s stepone Plus real-time PCR instrument with Tiangen’s SuperReal premix Plus. The relative expression levels were normalized to the internal control *PbActin*, *MdActin* and *NbActin* in *P. betulaefolia*, *M. domestica* and *N. benthamiana*, respectively. All primers used are listed in Table S1.

### 4.4. Extraction Protein of Plasmodesmata

The protein of plasmodesmata was extracted using the method described previously with little modification [56]. About 3 g leaf of *P. betulaefolia* was ground into powder in liquid nitrogen. 2 mL/g buffer A (100 mM Tris-HCl, 100 mM KCl, 10 mM EDTA, 0.45 M mannitol, 10% glycerin, pH = 8.0, 0.1 mM Phenylmethanesulfonyl fluoride (PMSF)) was immediately added, homogenized with the sample and filtered by nylon membrane with a pore size of 16 μm. The samples retained on the nylon cloth were collected. Then 2 mL/g buffer B (10 mM Tris-HCl, 100 mM NaCl, 10 mM EDTA, 10% glycerin, pH = 8.0, 0.1 mM PMSF) was added, mixed well, and filtered and collected with the 5 μm-pore nylon cloth at 4 °C. 30 mL buffer B was added to the samples retained on the nylon cloth for resuspension and ultra-sonication for 15 min. After 10,000× *g* centrifugation in a 50 mL RNase-free tube for 2 min at 4 °C, the supernatant was discarded. The sediment is resuspended with buffer B in ice bath, followed by 10,000× *g* centrifugation for 2 min at 4 °C. This process was repeated five times until the supernatant was no longer green. The precipitate after the last centrifugation was resuspended with buffer B in ice bath, and then filtered by a 5 μm-pore nylon cloth. The samples retained on the nylon cloth was mixed with digestion buffer C (10 mM MES, pH = 5.5, 240 mM mannitol), followed by a 400× *g* centrifugation at 4 °C for 5 min. The precipitate was resuspended into digestion buffer D (10 mM MES, pH = 5.5, 240 mM mannitol, 0.7% cellulase) and incubated at 55 °C for 5 min. After filtered with 2 μm nylon, the samples retained on the nylon cloth was resuspended with Buffer D and incubated in a 100 rpm shaker at 37 °C for 1.5 h. Next, the resuspension was centrifuged at 5850× *g* at 4 °C for 5 min. Finally, the supernatant was centrifuged at 110,000× *g* at 4 °C for 40 min and deposited at −80 °C for future use.

### 4.5. Co-Immunoprecipitation/Mass Spectrometry (CO-IP/MS)

The coding sequence of *PbANK* was amplified by PCR using primers listed in the Table S1, and subcloned into the pMD19-T vector (Takara) for further vector construction. For Co-IP/MS analysis, the coding region of *PbANK* was inserted into the SUPER1300-cFLAG vector with *HindIII* and *KpnI* restriction sites. The plasmid was transformed into *Agrobacterium* EH105 strain. The *Agrobacterium* was infiltrated into the leaves of four weeks old *P. betulaefolia* to express PbANK-FLAG. *P. betulaefolia* was cultured in dark for 24 h and then in 16 h/8 h (light/dark) photoperiod for 2 days. The leaves of *P. betulaefolia* were used to extract plasmodesmata proteins. Co-IP analysis was performed according to the manufacturer’s protocol (Bimake Bio). Briefly, the extraction of plasmodesmata proteins was incubated with anti-FLAG magnetic beads. The beads were washed with washing buffer twice and eluted with elution buffer. The elution was analyzed by mass spectrometry (College of Biology, China Agricultural University).

### 4.6. Yeast Two-Hybrid Assay

The coding sequences of T10 and *PbANK* were constructed on pGADT7 and pGBKT7 removing the stop codon, respectively. PbANK-AD and T10-BK were co-transferred into the AH109 (Weidi Bio) yeast strain using the PEG/LiAc method following the manufacturer’s protocol (Clontech). This transformed yeast was cultured on the DDO (SD/-Leu/-Trp) medium for three days, and then transferred to TDO (SD/-His/-Leu/-Trp) medium. The growth state of yeast on TDO (SD/-His/-Leu/-Trp) medium indicated that they interacted with each other or not. pGBK-P53 and pGAD-SV40 was used as the positive control, while the empty pGADT7 and pGBKT7 vectors were used as the negative control.

### 4.7. Bimolecular Fluorescence Complementation (BiFC) Assays

The coding sequences of *PbANK* and *T10* were inserted into the pCAMBIA1300-YFPc and pCAMBIA1300-YFPn vectors, respectively. The obtained vectors expressing PbANK-YFPc and T10-YFPn were transformed into *Agrobacterium* GV3101 strain. The cultures containing PbANK-YFPc and T10-YFPn were harvested and resuspended in infiltrated buffer (10 mM MgCl_2_, 0.2 mM acetosyringone and 10 mM 2-(4-morpholino) ethanesulfonic acid (MES), pH 5.6) at the concentration of OD_600_ = 1.0. The two cultures were mixed with a ratio of 1:1 and infiltrated into the leaves of *N. benthamiana* for transient expression. The pCAMBIA1300-YFPn and pCAMBIA1300-YFPc empty vectors were used as the negative control. The fluorescence level of YFP was observed at 48 h post infection (hpi) under the confocal microscope (Nikon A1) [2].

### 4.8. Luciferase Complementation Imaging (LCI) Assays

The coding sequences of *T10* and *PbANK* were inserted into the pCAMBIA1300-nLuc and pCAMBIA1300-cLuc vectors, respectively, and transformed into the *Agrobacterium* GV3101 strain. The infiltrated process was performed as described above. At 48 hpi, the leaves were infiltrated with an appropriate amount of 1 mM luciferase substrate (Beettle luciferin) and incubated in dark for 5 min. The LUCK2019 in vivo imaging system (LB985) was used for fluorescence image detection.

### 4.9. Subcellular Co-Localization

The coding sequences of T10 and *PDLP5* were inserted into pBI121-mCherry and Super1300-cGFP, respectively. The two cultures of *Agrobacteria* harboring *PbPDBG-mCherry* and *PDLP5-GFP* were co-infiltrated into the leaves of *N. benthamiana* for transient expression. The fluorescence of mCherry and GFP were observed using excitation at 594 nm and 488 nm, respectively, under confocal microscope (Nikon A1) at 72 hpi.

### 4.10. β-1,3-Glucanase Activity Assay

Crude enzyme extract: 1 g of tobacco leaves were collected and ground with liquid nitrogen, Add 0.1 mol/L pH 6.5 phosphate buffer solution (containing 5 mmol/L mercaptoethyl alcohol, 0.1 mmol/L EDTA and 0.5 mmol/L PMSF) and extract for 2 h. After centrifugation at 10,000× *g* for 20 min (4 °C), the supernatant was crude enzyme solution.

Enzyme activity: Take 0.1 mL of crude enzyme solution, add 0.5 mL of dextran storage solution (250 mg of laminarin dissolved in 50 mL of deionized water), 0.4 mL dinitrosalicylic reagent (Add 1.0 g of 3,5-dinitrosalicylic acid in 10 mL of distilled water), then add 1.6 g of sodium hydroxide and 30 g of potassium sodium tartrate, heat appropriately, and diluted with deionized water to 100 mL, mix well, keep at 37 °C for 1.5 h, then add 1.0 mL of salicylic acid storage solution and heating for 5 min in a boiling water bath to stop the reaction, immediately cool, add 3 mL of water to vortexed and its absorbance at 500 nm was determined.

The method for determining enzyme activity refers to Sundararaj and Kathiresan [57].

### 4.11. Bombardment

The vector pCAMBIA1305-GFP-PbPTB3 constructed previously [3] was used here to observe the intercellular diffusion efficiency. The leaf of *N. benthamiana* moistening the petiole with paper was placed in a petri dish under the bombardment device. Then 10 μL pCAMBIA1305-GFP-PbPTB3 plasmid (1000 μg/μL) vortexed with 4 μL spermidine (100 mM), 10 μL CaCl_2_ (2.5 M) and 6 μL gold particles for 10 min, followed by rinsing with 70% ethanol twice and resuspension in pure ethanol. The gold particles with plasmid were bombarded into the leaf of *N. benthamiana*. The bombarded leaves were kept under moisture for 48 h for transient expression. The fluorescence of GFP was observed under confocal microscope (Nikon A1). The number of cells with fluorescence migration was counted. The statistical analysis was performed using Prism 8 software.

### 4.12. Callose Staining

The leaves of *N. benthamiana* were fixed with ethanol solution containing 10% glacial acetic acid for more than 1.5 h or overnight and further soaked in 1 M NaOH overnight. Then the leaves were wash with 50 mM potassium metaphosphate for 3 times. Finally, the leaves of *N. benthamiana* were stained with 200 uL 0.01% aniline blue for 10 min and placed on slides to observe callose fluorescence under laser confocal microscope (Nikon A1). The content of callose in tobacco leaves under different treatments was quantified. The further statistical analysis was performed using Prism 8 software.

### 4.13. dCAPS Analysis

The dCAPS analysis was performed using the method described previously [12], using the specific *SfcI* digestion site between *PbWoxT1* and *MdWoxT1* with a G/A SNP site at 565 bp of both *WoxT1* mRNA. A 735 bp PCR product was cloned from cDNA of *P. betulaefolia* and *M. domestica* with nested PCR primer listed in Table S1. The obtained PCR products were purified and digested with *SfcI* restriction endonuclease. The *PbWoxT1* product could be cleaved into two sections of 565 bp and 170 bp, while the *MdWoxT1* show a 735 bp band without being cleaved. All products were separated on a 2% agarose gel. By the size of the cleaved fragment, *WoxT1* in stock and scion could be distinguished from *P. betulaefolia* or *M. domestica*.

### 4.14. The Transformation of Tobacco

To generate the *PdPDBG* overexpression line (OE-*PbPDBG*), *PbPDBG* coding sequence was inserted in the *pFGC5941* vector, promoted by *CaMV 35S* promoter. To construct the *PDBG* silencing (*PDBG* RNAi) vectors, about 200 bp sense and antisense fragments were inserted in the *pFGC5941* vector on either side of the intron for expression driven by the *CaMV 35S* promoter. The two vectors were transformed into *Agrobacterium* GV3101 strain to transform tobacco (*Nicotiana benthamiana* and *Nicotiana tabacum* var. Wisconsin 38). The genetic transformation of tobacco uses the method previously described [58]. The *PbANK* overexpression and silencing tobacco lines were created previously [3]. All primers used were listed in Table S1.

### 4.15. The Transient Expression of P. betulaefolia

To construct the vector for the transient expression in *P. betulaefolia*, *PbANK* was inserted into the *pCAMBIA1305.1* vector [59] promoted by *CaMV 35S* for its overexpression. Virus-induced gene silencing (VIGS) system was used to silence *PbANK* [60]. About 200 bp fragment was inserted into the *pTRV2* vector promoted by *CaMV 35S*. *pTRV1*, *pTRV2*-*PbANK* and *pTRV2* empty vector was transformed in the *Agrobacterium* GV3101 strain. The *pTRV1* cells were then mixed in a 1:1 ratio with that containing either *pTRV2*-*PbANK* or *pTRV2* empty vector. The transformed cells were co-infiltrated into *P. betulaefolia* under a vacuum of 65 kPa for 20 min. The transformed plantlets were cultivated at 25 °C for 3 d and then analyzed by western-blot. All primers used were listed in Table S1.

## 5. Conclusions

In this study, we identified a novel β-1,3-glucanase PbPDBG protein in pear, which played a role in callose deposition. PbANK interacted with and recruited PbPDBG to plasmodesmata. PbPDBG itself was not mobile and degraded callose at plasmodesmata in situ to facilitate the long-distance transport of *PbWoxT1* and PbPTB3 formed ribonucleoprotein complexes. Our findings provide the basis to further study the ribonucleoprotein complex transport in pear and other species.

## Figures and Tables

**Table 1:** The transport related proteins interacting with PbANK screened out by Co-IP.

NO.	Description
T1	Glycerate dehydrogenase-like protein
T2	Carbonic anhydrase isoform 1
T3	Ferritin OS = Pyrus x bretschneideri
T4	Pgip protein
T5	Fructokinase
T6	Monodehydroascorbate reductase
T7	Glutathione peroxidase
T8	Spermidine synthase
T9	Ascorbate peroxidase
T10	Beta-1,3-glucanase
T11	Aspartate aminotransferase

## Data Availability

The data presented in this study are available in article or supplementary material here.

## Supplementary Materials

The following supporting information can be downloaded at: https://www.mdpi.com/article/10.3390/ijms24098051/s1.

## Abbreviations

*PbWoxT1*	Wuschel-related homeobox transport1
PDBG	Plasmodesmata-localized β-1,3-glucanase
PTB	polypyrimidine tract binding
ANK	ankyrin repeat domain
*TTG1*	Transpareet testa glabra1
*GAI*	Gibberellic acid insensitive
BiFC	Bimolecular fluorescence complementation
RBP	RNA-binding protein
VIGS	Virus-induced gene silencing
