# Supplementary material for: The β-1,3-Glucanase Degrades Callose at Plasmodesmata to Facilitate the Transport of the Ribonucleoprotein Complex in Pyrus betulaefolia
Source: Int J Mol Sci. 2023 Apr 29;24(9):8051. doi: 10.3390/ijms24098051 (PMC10179145; doi:10.3390/ijms24098051)
Supplement: Supplementary file 1 [file ijms-24-08051-s001.zip › ijms-2317900-supplementary.pdf]

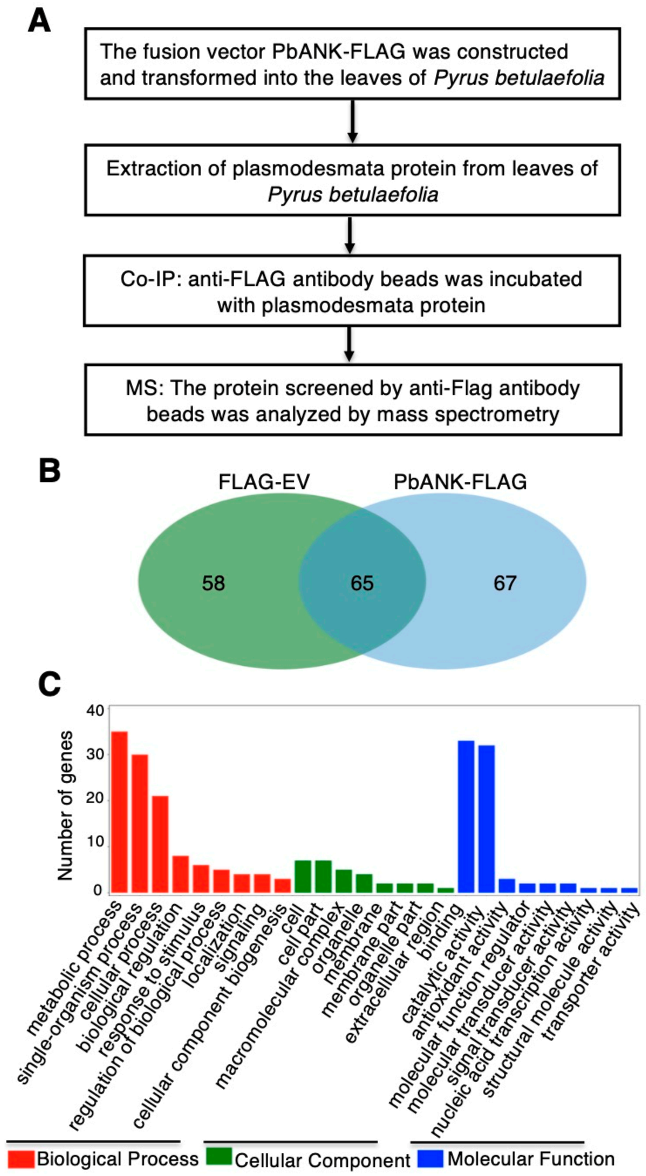

Figure S1. Exploration of PbANK binding proteins at plasmodesmata (PD). **(A)** The flow chart depicted the PbANK-bound proteins at PD obtained by Co-IP/MS assay. **(B)** Mass spectrometric analysis showed that the protein obtained from the PD of *P. betulaefolia* transfected with the PbANK-FLAG fusion vector or the FLAG empty vector. The interacting proteins were co-precipitated by Anti-FLAG beads. The Venn diagram highlighted 67 proteins identified as specific interaction partners of PbANK-FLAG. **(C)** GO enrichment analysis of the 67 proteins interacting with PbANK (FDR<0.01).

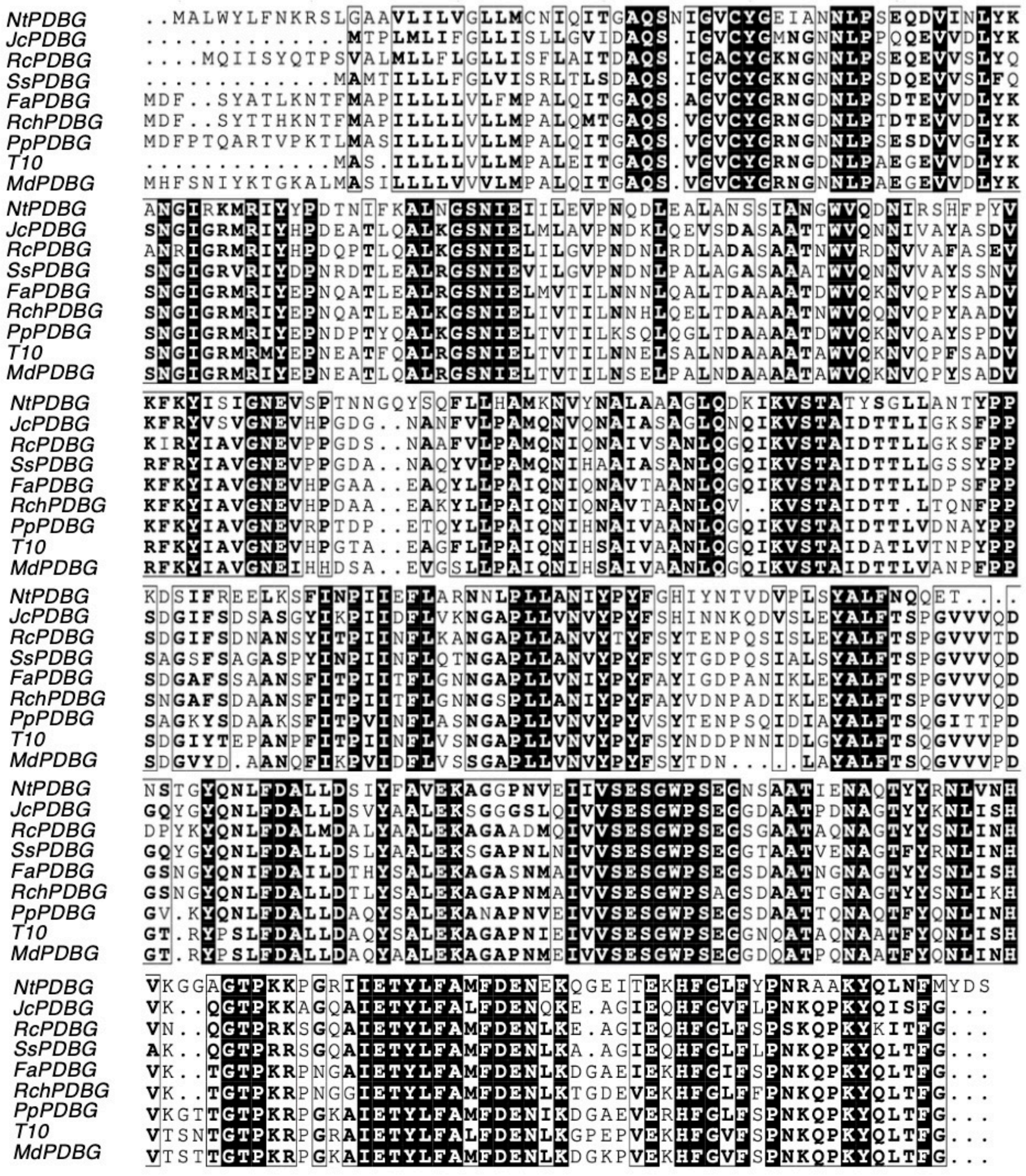

Figure S2. Aligning amino acid sequence of T10 genes from different species. The column on the left represented the β-1,3-glucanase genes from different species. The black box segment indicates the part with high homology of β-1,3-glucanase among species. T10 indicated the homology gene from *P. betulaefolia* which we identified.

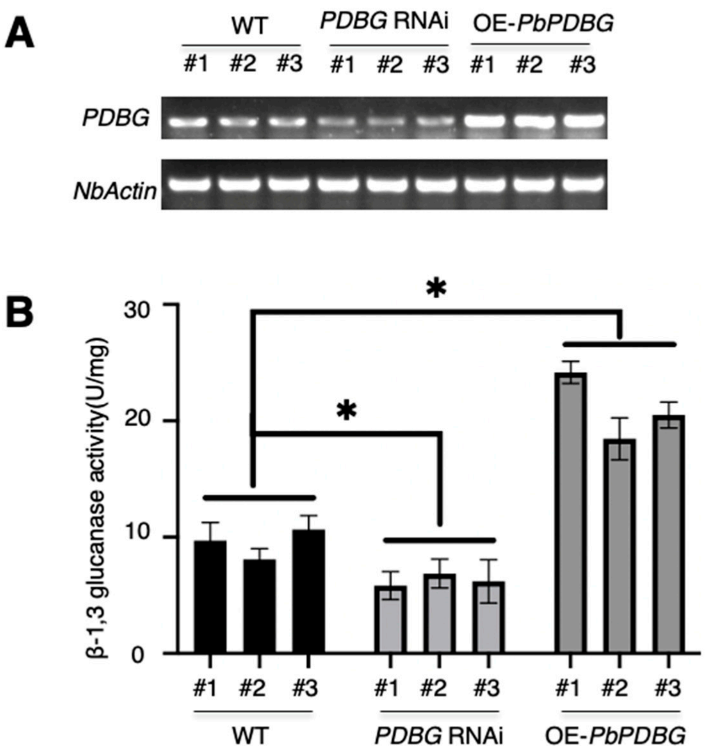

Figure S3. Determination of PDBG expression and β-1,3 glucanase activity in PDBG transgenic tobacco lines. **(A)**The RT-PCR analysis of *PbPDBG* expression level in wild type (WT), the *PbPDBG* overexpression line (OE-*PbPDBG)* and the *PDBG* RNAi line of N. benthamiana. *NbActin* was used as internal control. #1, #2, and #3 represented three biological replicates. **(B)** The β-1,3 glucanase activities of OE-*PbPDBG*, *PDBG* RNAi and wild-type (WT) leaves of N. benthamiana were determined respectively. #1, #2, and #3 represented three biological replicates.

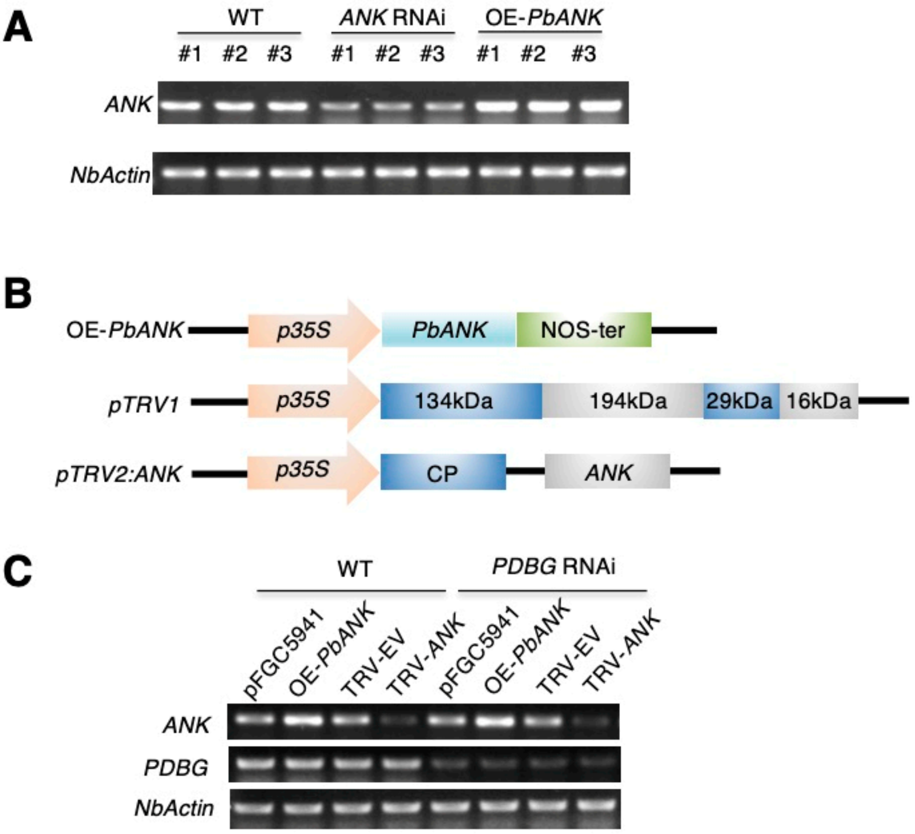

Figure S4. The expression level of *ANK* and *PDBG*. **(A)** The RT-PCR analysis of *PbANK* expression level in wild-type, ANK silencing (*ANK* RNAi) and *PbANK* overexpression (OE-*PbANK*) lines. *NbActin* was used as internal control. #1, #2, and #3 represented three biological replicates. **(B)** The vector diagrams of OE-*PbANK*, *TRV1* and *TRV2:ANK* used for over-expression or silencing of *PbANK*. **(C)** The RT-PCR analysis of *PDBG* and *ANK* expression level in wild type (WT) and *PDBG* RNAi *N. benthamiana* co-overexpressed *PbANK* (OE- *PbANK*) and co-silenced *ANK* (TRV-*ANK*). *NbActin* was used as internal control.

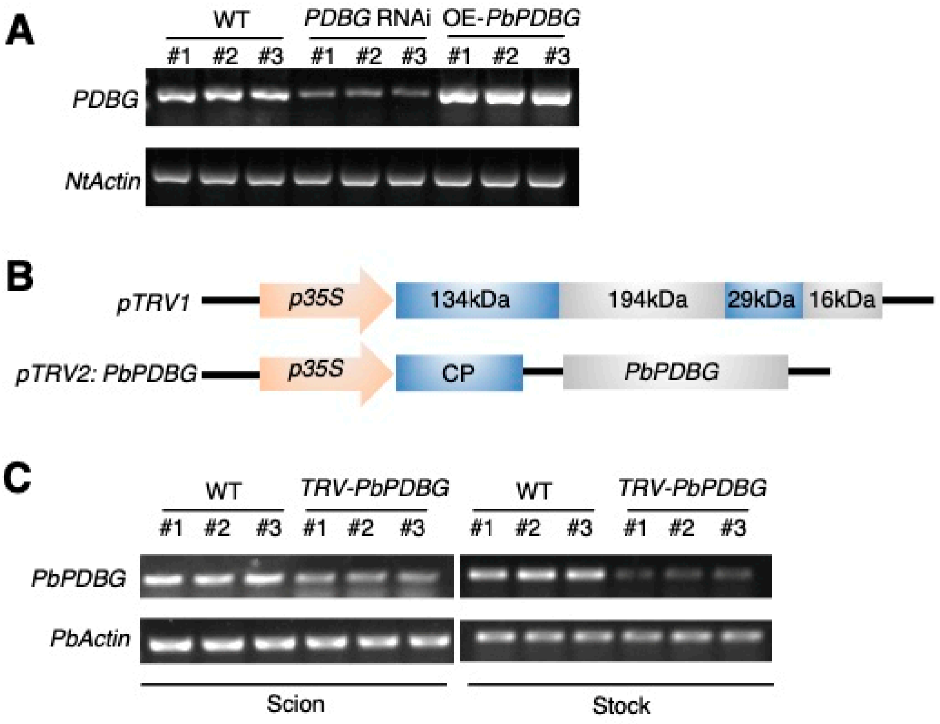

Figure S5. The expressing analysis of *PDBG*. **(A)** The RT-PCR analysis of *PDBG* expressing level in wild type (WT), the *PbPDBG* overexpression line (OE- *PbPDBG*) and *PDBG* RNAi lines used in grafting experiments. *NtActin* was used as internal control. **(B)** The vector diagrams of *TRV1* and *TRV2: PbPDBG* used for silencing of *PbPDBG*. **(C)** The RT-PCR analysis of *PbPDBG* expressing level in wild type (WT) and *TRV- PbPDBG* used in grafting experiments. *PbActin* was used as internal control. In (A) and (C), #1, #2, and #3 represented three biological replicates.

Table S1. Primers used in the article

Primer	sequence
Flag-PbANK-F	ATCGACTCTAGAAAGCTTATGGCTTCCACGCAGAAGGAT
Flag-PbANK-R	TTTGTAGTCCATGGTACCCAGGAAGGCGTCCTTCTCG
HA-PbPDBG-F	GAGAACACGGGGGACTCTAGAATGGCTTCCATATTGCTACTACTTGTAC
HA-PbPDBG-R	GCCCCCGGGGTCGACGGTACCTCCGAAGGTGAGTTGGTACTTGTG
pGADT7 -PbANK-F	ATGGAGGCCAGTGAATTCATGGCTTCCACGCAGAAGGAT
pGADT7 -PbANK-R	CTCGAGCTCGATGGATCCACAGGAAGGCGTCCTTCTCG
pGBKT7-PbPDBG-F	GAGGAGGACCTGCATATGATGGCTTCCATATTGCTACTACTTGTAC
pGBKT7-PbPDBG-R	CTGCAGGTCGACGGATCCTCCGAAGGTGAGTTGGTACTTGTG
YFPc-pbANK-F	ATCTATCTCTCTCGAGGTACCATGGCTTCCACGCAGAAGGAT
YFPc-pbANK-R	ATCTATCTCTCTCGAGGTACCATGGCTTCCACGCAGAAGGAT
YFPn-PbPDBG-F	ATCTATCTCTCTCGAGGTACCATGGCTTCCATATTGCTACTACTTGTAC
YFPn-PbPDBG-R	GTCGACTCCGAATTCGGATCCTCCGAAGGTGAGTTGGTACTTGTG
cluc-PbANK-F	GTCGACTCCGAATTCGGATCCTCCGAAGGTGAGTTGGTACTTGTG
cluc-PbANK-R	ACGAAAGCTCTGCAGGTCGACCAGGAAGGCGTCCTTCTCG
nluc-PbPDBG-F	ACGGGGGACGAGCTCGGTACCATGGCTTCCATATTGCTACTACTTGTAC
nluc-PbPDBG-R	CGCGTACGAGATCTGGTCGACTCCGAAGGTGAGTTGGTACTTGTG
mcherry-PbPDBG-F	AACACGGGGGACTCTAGAATGGCTTCCATATTGCTACTACTTGTAC
mcherry-PbPDBG-R	CTTGCTCACCATGGTACCTCCGAAGGTGAGTTGGTACTTGTG
PDLP5-GFP-F	CTCTAGTCTAGAAAGCTTATGATCAAGACAAAGACGACGTCCC
PDLP5-GFP-R	GGTACCGGATCCACTAGTTTGCCCTTCTCTCCTTTCATGACC
RT-PbPDBG-F	CGCAACTCTTGTGACCAACC
RT-PbPDBG-R	ACCACGACCCCTTGTGAAGT
RT-NtPDBG-F	ACAAGATGGTTCCCGCCAAT
RT-NtPDBG-R	TCCAGGCTTTCTTGGGCTAC
NtPDBG RNAi-F-1	TTACATTTACAATTACCATGGTACAAGATGGTTCCCGCCAA
NtPDBG RNAi-R-1	GAAATTCTTACACATTTAAATTGGGCTACCCTCTTTAGCGT
NtPDBG RNAi-F-2	TGGATCCTAGGTGAGTCTAGATGGGCTACCCTCTTTAGCGT
NtPDBG RNAi-R-2	ACTCTAGGGACTAGTCCCGGGTACAAGATGGTTCCCGCCAA
OE-NtPDBG-F	GCCCAATCGATGATTTAAATATGCTAGGCAACAACTTGCCAA
OE-NtPDBG-R	CTAGGGACTAGTCCCGGGTATTTGGGCTGCTTGTTGGGG
TRV-PbANK-F	ACGCGTGAGCTCGGTACCGCGAAAAGCCAGTTCCAACA
TRV-PbANK-R	GTTACCGAATTCTCTAGATACGTGACATGCGTTCCTCAA
OE-PbANK-F	GCCCAATCGATGATTTAAATATGGCTTCCACGCAGAAGGAT
OE-PbANK-R	CTAGGGACTAGTCCCGGGCAGGAAGGCGTCCTTCTCGA
pEZS-PbPDBG-F	GAGACTCGAGATGGCTTCCATATTGCTACTACTTGTAC
pEZS-PbPDBG-R	GAGAGGATCCTCCGAAGGTGAGTTGGTACTTGTG
TRV-PbPDBG-F	GTTACCGAATTCTCTAGACACTTCACAAGGGGTCGTGGT
TRV-PbPDBG-R	ACGCGTGAGCTCGGTACCCCAGGCCTCTTTGGAGTCCC
RT-PbWoxT1-F	TCGGCAAGATCGAAGGCAAGA
RT-PbWoxT1-R	CACACCAACTGAAGAAGAAGATG
pEZS-PbPTB3-F	GAGACTCGAGATGACAGAACCTTCTAAAG
pEZS-PbPTB3-R	GAGAGGATCCATTGCCTGTAGCTGCGAG
RT-NtANK-F	GAGGCGAGAACAACCTCCAC
RT-NtANK-R	GAAGTTGCTCCGCCATCTGA
RT-PbANK-F	GAGTTTCGCCAATCCATCAAAC
RT-PbANK-R	GCTGGACCACCAGTTTCTATT

List of Abbreviations

Abbreviation	Full name
**BiFC**	Bimolecular fluorescence complementation
**CBB**	Coomassie brilliant blue
**Co-IP**	Co-immunoprecipitation
**EDTA**	Ethylene diamine tetraacetic acid
**GAI**	Gibberellic acid insensitive
**LCI**	Luciferase complementation imaging
**MS**	Mass Spectrometry
**PbANK**	Pyrus betulaefolia Ankyrin repeat-containing protein
**PbPTB3**	Pyrus betulaefolia polypyrimidine tract–binding
**PbWoxT1**	Pyrus betulaefolia Wuschel related homeobox
**PD**	Plasmodesmata
**PDBG**	Plasmodesmata-localized β-1,3-glucanase
**PDLP5**	Plasmodesmata-located protein 5
**RNP**	ribonucleoprotein
**RT-PCR**	Reverse transcriptase PCR
**SEL**	Size of exclusion limit
**SNP**	Ethylmethylsulfone
**SNP**	Simple Nucleotide Polymorphism
**TMV**	Tobacco mosaic virus
**VIGS**	VIGS Virus-induced gene silencing
